# Estimating the prevalence of text overlap in biomedical conference abstracts

**DOI:** 10.1186/s41073-020-00106-y

**Published:** 2021-02-01

**Authors:** Nick Kinney, Araba Wubah, Miguel Roig, Harold R. Garner

**Affiliations:** 1 Edward Via College of Osteopathic Medicine, 2265 Kraft Drive, Blacksburg, VA 24060 USA; 2 Gibbs Cancer Center & Research Institute, Spartanburg, SC USA; 3 St. John’s University, 300 Howard Avenue, Staten Island, NY 10301 USA

**Keywords:** Text similarity, Plagiarism, Duplication, Salami publication, Conference presentations

## Abstract

**Background:**

Scientists communicate progress and exchange information via publication and presentation at scientific meetings. We previously showed that text similarity analysis applied to Medline can identify and quantify plagiarism and duplicate publications in peer-reviewed biomedical journals. In the present study, we applied the same analysis to a large sample of conference abstracts.

**Methods:**

We downloaded 144,149 abstracts from 207 national and international meetings of 63 biomedical conferences. Pairwise comparisons were made using eTBLAST: a text similarity engine. A domain expert then reviewed random samples of highly similar abstracts (1500 total) to estimate the extent of text overlap and possible plagiarism.

**Results:**

Our main findings indicate that the vast majority of textual overlap occurred within the same meeting (2%) and between meetings of the same conference (3%), both of which were significantly higher than instances of plagiarism, which occurred in less than .5% of abstracts.

**Conclusions:**

This analysis indicates that textual overlap in abstracts of papers presented at scientific meetings is one-tenth that of peer-reviewed publications, yet the plagiarism rate is approximately the same as previously measured in peer-reviewed publications. This latter finding underscores a need for monitoring scientific meeting submissions – as is now done when submitting manuscripts to peer-reviewed journals – to improve the integrity of scientific communications.

**Supplementary Information:**

The online version contains supplementary material available at 10.1186/s41073-020-00106-y.

## Introduction

Although reliable dissemination of research is critical to the advancement of knowledge, the past 3 to 4 decades have witnessed growing concerns over the integrity of the scientific and scholarly record. In response, governments [1–4] and research institutions across the world [5, 6] have developed guidance for avoiding ethically questionable practices and, especially, the more serious forms of research misconduct.

A common research misbehavior that often rises to the level of research misconduct is plagiarism. Generally defined as passing off the work of others as one’s own [7], plagiarism can manifest itself in many forms. Unfortunately, authors and even journal editors seem to differ in terms of how much text overlap is acceptable, whether from one’s prior work (i.e., text recycling) or from others’ work (i.e., plagiarism) [8–12]. Even conceptions of plagiarism can differ widely amongst this latter group of professionals [13].

A related ethically questionable practice frequently included in discussions of plagiarism is self-plagiarism, which generally refers to an author’s reuse of his/her previously disseminated work as new content. Even when the amount of reuse is deemed excessive, most self-plagiarism of text (commonly known as text recycling) does not meet the definition of research misconduct [14]. According to the Committee on Publication Ethics (COPE) guidelines, editors are encouraged to use their own judgment to decide how much text overlap is acceptable in situations in which some reuse of textual content may be unavoidable [15]. Nonetheless, editors report that plagiarism, text recycling, and other forms of overlap, such as duplicate publication (i.e., publishing a paper in a journal that is essentially the same paper that had been previously published in a different journal), are some of the most frequent problems they encounter [16]. Together, these lapses contribute to a substantial percentage of the retracted literature [17–20]. For example, a check of the Retraction Watch database [21], shows that of the 23,863 papers recorded as having been retracted as of August 11, 2020, a total of 2464 (10%) had been retracted for plagiarism. However, just as there is no all-embracing definition of textual plagiarism, there is also no agreed-upon operational definition of text recycling [11, 22].

Evidence from other sources supports the contention that plagiarism is a persistent problem in science and scholarship. For example, in addition to journal editors’ complaints that a significant number of submissions to their journals contain plagiarized content [23–28], surveys of scientists have also consistently revealed that some admit to having engaged in some form of plagiarism [29, 30]. For example, an analysis of published studies on the subject carried out by Pupovac and Fanelli suggests that 1.7% of scientists admit to committing plagiarism and 30% admit to witnessing plagiarism [30]. But, such surveys likely underestimate the extent to which respondents commit this type of transgression given evidence that some academics appear to be unfamiliar with traditional scholarly conventions, (e.g., proper citations and paraphrasing) [8] and, therefore, may be plagiarizing inadvertently.

Using text analytics we pioneered [31–33] and rigorously applied to Medline/Pubmed [34–37], we have shown that peer-reviewed publications contain 0.4% duplicate publications with different authors (plagiarism) and 1.35% duplicates (self-plagiarism) with the same authors [38]. The rate of false positives in that study was 1%. Using an analogous text analytic procedure, with somewhat different criteria for determining textual duplication, at least one other large-scale study of actual manuscripts (which are not peer-reviewed) from the entire arXiv pre-print server has found even greater amounts of textual overlap [39].

Indeed, while the presence of plagiarism in scientific publications has been well-documented, this type of misbehavior in conference meetings has received limited attention. One early study of conference submissions reported that self-plagiarism, rather than plagiarism, was the basis for most of the textual overlap reported [40]; another study of actual conference papers revealed roughly the opposite outcome [41]. Two other studies of papers presented at conferences in the field of Management have reported considerable amounts of textual overlap [42, 43].

Hundreds of thousands of scientists attend and make presentations at thousands of national and international meetings each year – at a likely cost of millions of dollars annually. Given the extent to which plagiarism and self-plagiarism occur in the print literature and the possibility that the conference environment presents similar, if not greater, possibilities for these exchanges to violate known established scientific norms, we initiated the present investigation. Our study was grounded by the informal observation that while guidance on research integrity is stated by most scholarly journals [44], ethical standards for scientific conferences (e.g., cautioning authors against plagiarism) are vague or sometimes not explicitly stated but only implied upon submission of scientific findings. Moreover, with some exceptions [41, 45], this form of scientific exchange does not appear to be as heavily screened for integrity issues as journal papers are [12]. In view of growing concerns about the integrity of the scientific record and the need for transparency in all aspects of research, we attempted to estimate the extent of text overlap in a large sample of conference abstracts spanning several meetings across various disciplines in the biomedical sciences. Our general hypothesis was that comparable levels of text overlap as those observed in prior studies with Medline abstracts would be observed in scientific meetings.

## Methods

### Primary data curation and availability

Conference proceedings were identified online using search terms such as “biomedical conferences”, “medical conferences”, “biomedical abstracts” and “medical abstracts”. We used google advanced search: https://www.google.com/advanced_search/. Proceedings in PDF format were identified by searching against filetype. Only conference abstracts were considered for analysis; we did not consider published abstracts. To be sure we only considered conference abstracts, each was extracted from manifests provided within documented conference proceedings (available on request).

Abstracts within each PDF and HTML document were extracted (scraped) using Python and stored in a MySQL relational database. The complete database of web-scraped abstracts is available online at www.ethicsdb.org (http://205.186.56.104/largeDatabase/) and, currently, contains 144,149 abstracts from 207 meetings of 63 conferences. In addition to conference abstracts, the database contains 327,287 published abstracts downloaded from MEDLINE. The content of www.ethicsdb.org was used as the primary source of data for the remainder of this study; the dedicated website includes a summary page, search page, export pages, and two results pages (details below). Additional details on the use and navigation of the database are given in a video (https://www.youtube.com/watch?v=3MH2WixtbIQ).

### Primary data curation limitations

Proceedings were initially identified for 63 different conferences; however, we were not able to identify proceedings for every meeting (iteration) of these conferences. We performed a focused search for each conference, which on average returned 3 previous yearly meetings (207 meetings of 63 conferences). We did not solicit conference organizers for abstracts or inquire if proprietary plagiarism checks are employed during submission. In addition, we only considered poster abstracts. Workshops, plenary talks, and keynotes were classified as false positives (see below).

### Pairwise abstract comparisons

**Fig. 1:**
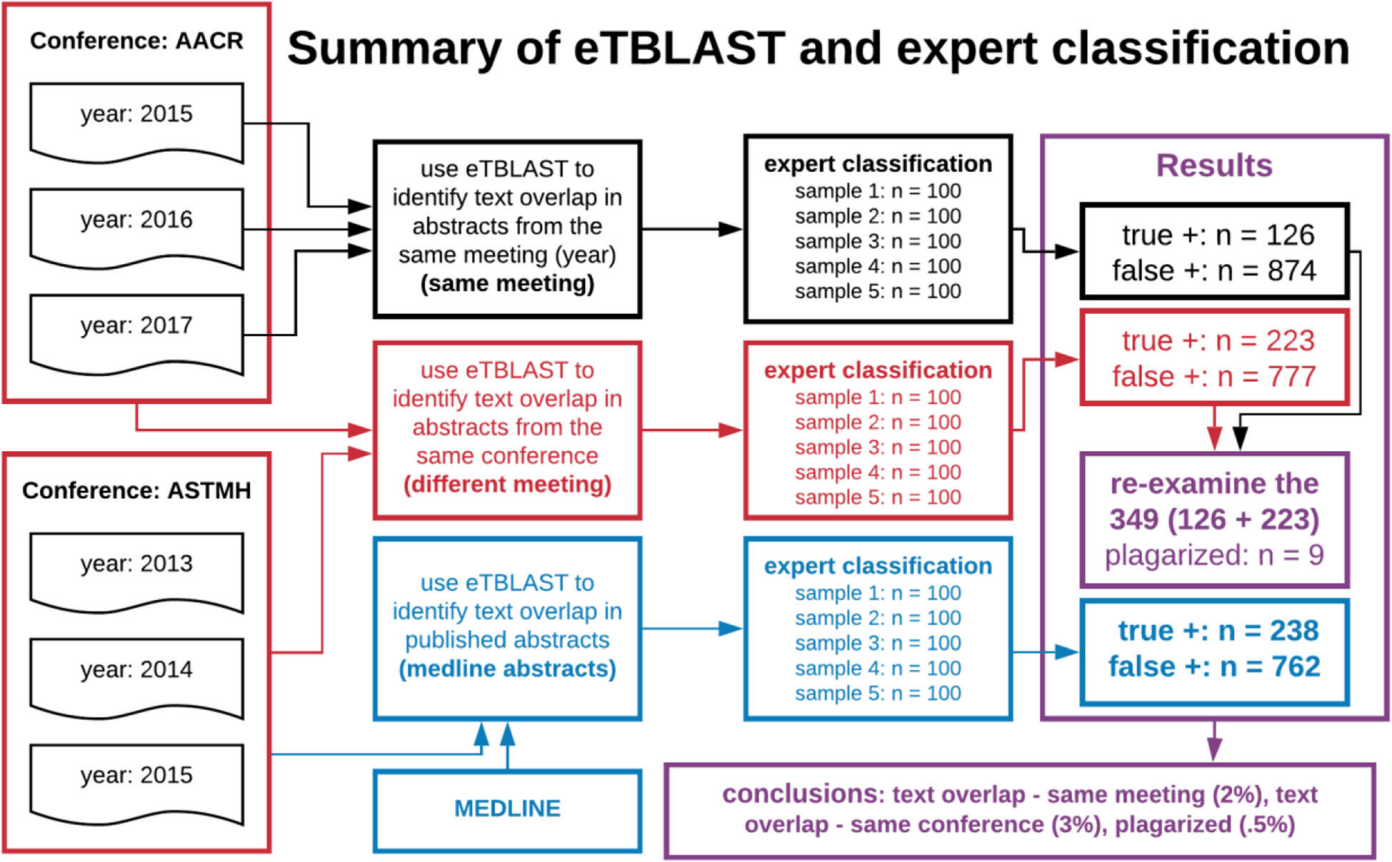
Summary of eTBLAST and expert classification. Our study was conducted from left to right. Far left, conference proceedings were web-scraped or pdf-scraped and deposited into a free online database: http://www.ethicsdb.org/. We considered 207 meetings of 63 different conferences. Middle left, we used eTBLAST to compare abstracts within each conference meeting (gray box), between each conference (red box), and to published abstracts in medline (blue box). Middle right, random samples of highly similar abstracts identified by eTBLAST were evaluated for misconduct by a domain expert (ethicist). Far right, random sampling and classification identified 126 (12.6%) instances of text overlap – same meeting, 223 (22.3%) instances of text overlap – same conference, and 238 (23.8%) instances of text overlap with MEDLINE. Bone-fide plagiarism was rare; we identified 9 potential instances. We concluded that text overlap – same meeting, text overlap – same conference, and plagiarized abstracts account for approximately 2, 3%, and .5% of conferences abstracts, respectively

On average, abstracts for each conference span 3 meeting years (207 meetings of 63 conferences). We performed a pairwise comparison of abstracts within each meeting and between each meeting; pairwise comparisons between meetings were only considered for different years of the same conference. In other words, we compared each meeting to itself and its previous occurrences (Fig. 1, far left). Pairs of abstracts considered in comparisons (within meetings and between meetings) were assigned similarity scores (details below). The top two similarity scores for each abstract in each meeting comparison are available at www.ethicsdb.org/view_scores.php. A summary of similarity scores is available for highly similar pairs at the level of 40%, 70%, and 90%, respectively. Note that Fig. 1 is a schematic that only shows two conferences with three meetings each; however, our approach draws samples from all 63 conferences and 207 meetings. Including all conferences in the same figure was unfeasible. An alternative schematic to further clarify our approach is provided in Fig. 3.

The second series of pairwise comparisons considered each collection of meeting abstracts and published abstracts downloaded from MEDLINE. The top three similarity scores for each abstract are available at www.ethicsdb.org/view_medline.php.

### Similarity scores, automated analysis with eTBLAST

Pairwise abstract similarity scores were assigned using eTBLAST (etblast.org), a text similarity engine (http://etblast.org/ A free service maintained by Heliotext LLC) inspired by BLAST [33–38], Details of eTBLAST can be found elsewhere [46–49]. Briefly, there are four steps in the eTBLAST algorithm: (a) removal of stop words and generation of keyword frequencies; (b) expansion of each keyword in the abstract to its set of lexical variants; (c) keyword search against a list of target abstracts; and (d) seed and extension of pairwise abstract alignments around matching keywords in both directions. Low similarity scores (approximately 0–.3) are indicative of two abstracts with few keywords in common; high similarity scores (approximately .5–1) are indicative of two abstracts with many keywords in common. A typical abstract in www.ethicsdb.org has 9 (sometimes more) similarity scores: the top two scores when compared to all other abstracts from the same meeting; the top two scores when compared to all other abstracts from two (sometimes more) previous yearly meetings (four or more total); and the top three scores when compared to published abstracts in MEDLINE (Fig. 1, middle left).

### Definition of highly similar abstracts and abstract pairs

We reiterate that similarity scores for pairs of abstracts were assigned with eTBLAST. The distribution of similarity scores over all pairwise comparisons was used to establish an objective definition of highly similar abstracts (see results). Briefly, eTBLAST assigned similarity scores to 1,235,085 abstract pairs. Approximately 2% (24,365 total) of pairwise abstract similarity scores exceeded .57 (2.5 standard deviations above the mean). By our definition, highly similar abstracts exceed this cutoff (see also results). This cutoff is arbitrary, but consistent with most statistical definitions of outliers. Abstracts with textual overlap were subsequently verified by a domain expert.

**Fig. 2:**
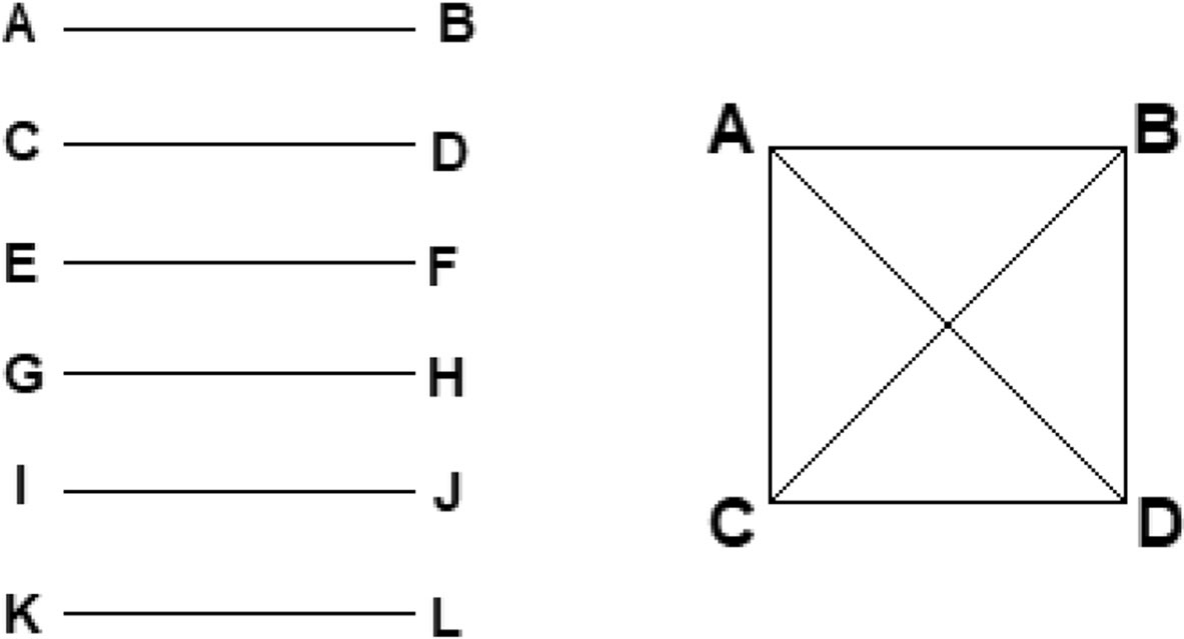
Pairs of similar abstracts are not trivially related to the number of abstracts within those pairs. Left, 12 abstracts can form 6 highly similar pairs. Right, 4 abstracts can form 6 highly similar pairs. In our work we used eTBLAST to make 1,235,085 pairwise comparisons. Approximately 2% (24,365 total) of those are highly similar pairs. Abstracts within highly similar pairs number 20,857. Apparently, there is non-trivial structure within the highly similar pairs of abstracts; however, assessing this structure is beyond the scope this work

It should be noted that the number of similar abstracts is not trivially related to the number of similar abstract pairs identified by eTBLAST. Most of the similar abstract pairs identified by eTBLAST are simple tandem pairs (Fig. 2, left). However, mutually overlapping pairs contain fewer abstracts than tandem pairs (Fig. 2, right). A hypothetical example is provided in Fig. 2. The lower bound for number of abstracts (n) within overlapping pairs is provided by binomial coefficient n!/2!(n^− 2^)!; i.e. n choose 2. The corresponding upper bound is simply 2n. Conceivably, this adds complexity to our approach, which randomly sampled abstract pairs for expert classification. However, most of the randomly sampled abstract pairs were simple tandem pairs; only a few were overlapping. It is very unlikely that mutually overlapping abstracts affected our results. It is beyond the scope of this work to characterize the structure of mutually overlapping similar abstracts within the collection of conferences and meetings selected for programmatic (eTBLAST) and expert analysis.

### Definition of text overlap classifications

We ran eTBLAST multiple times for each conference meeting (Fig. 1, far left): once to compare abstracts within the meeting; twice (or more) to compare meeting abstracts with from previous years of the same meeting, and once to compare meeting abstracts to published abstracts in MEDLINE. Random samples of highly similar abstracts (defined above) were subsequently used to quantify four types of misconduct defined below:

- *Text Overlap – Same Meeting.* Highly similar abstracts (similarity score exceeding .57) presented concurrently at the same meeting with at least one overlapping author. See Fig. S1 for example.
- *Text Overlap – Different Meetings*. Highly similar abstracts presented at different meetings (two different years) of the same conference with at least one overlapping author. See Fig. S2 for example.
- *Text Overlap – Medline Abstracts*. Conference abstracts highly similar to abstracts from Medline of previously published journal articles. Here, we made sure that the publication date in MEDLINE preceded the conference presentation date. In addition, these abstracts shared at least one overlapping author. We emphasized that in many cases it is acceptable to present abstracts that are already published. See Fig. S3 for example.
- *Plagiarized – Medline Abstracts*. Highly similar abstracts – but no overlapping authors – concerning any other conference abstract or previously published abstract in MEDLINE.
- *False positives.* Fringe cases that are not deemed misconduct by an ethicist. These also include abstracts with possible input errors arising from web-scraping and PDF-scraping. See Fig. S5 for example.

### Abstract classification

**Fig. 3:**
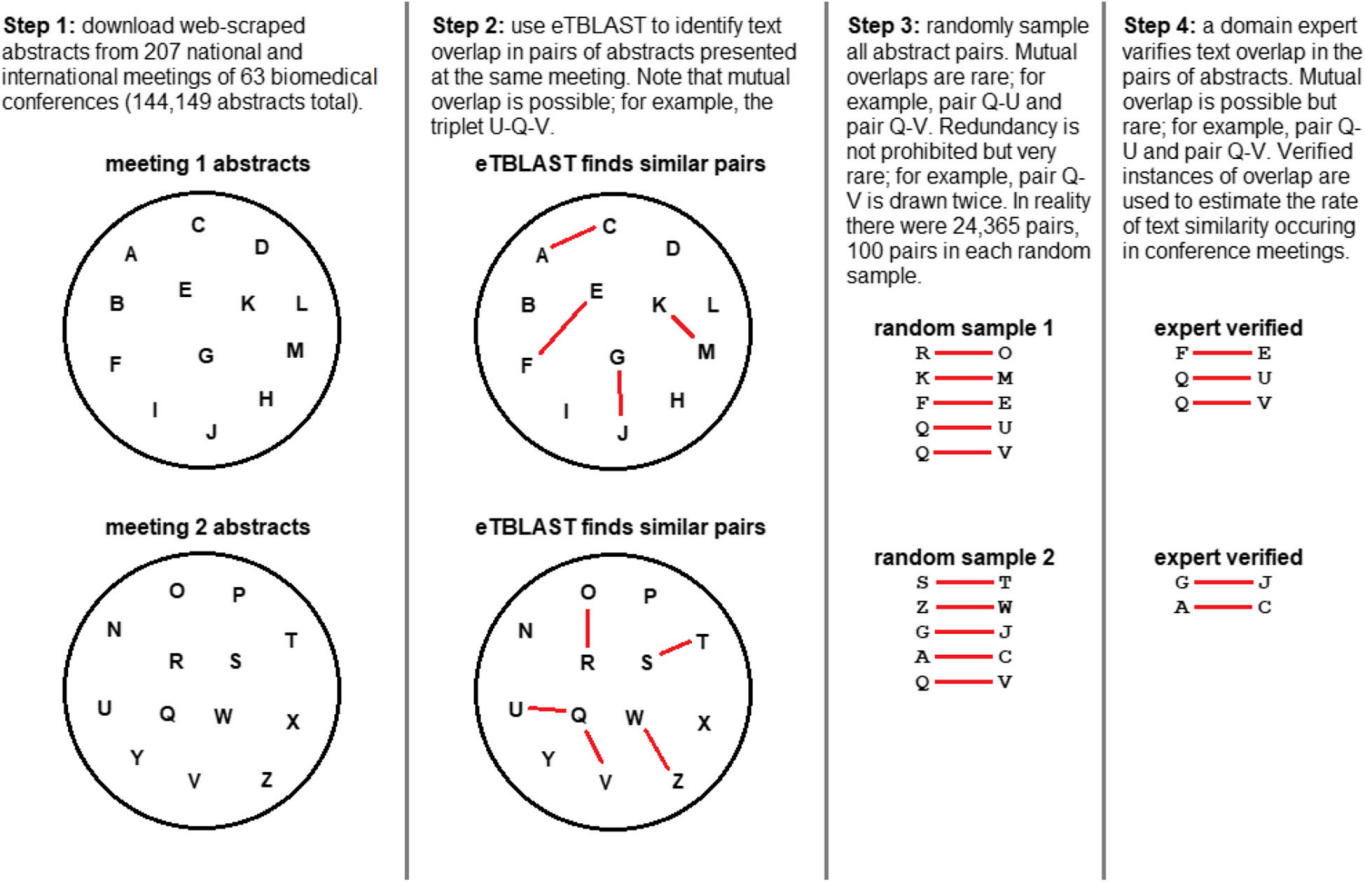
We illustrate our approach by considering a hypothetical collection of abstracts belonging to two different meetings (far left). To quantify text overlap (same meeting) we use eTBLAST to assign similarity scores between pairs of abstracts withing those two meetings (middle left). Mutual overlap is possible; for example, there is a triplet U-Q-V of mutual text overlap in the hypothetical meeting 2 (middle left). Next, we randomly sample abstract pairs for review. Here, mutual overlap is possible but rare; for example, pair Q-U and pair Q-V in random sample 1 (middle right). Thus, 1 abstract could be compared with 2 or 3 possible overlaps, but rarely. Verified abstracts are used to estimate the rate of text similarity (within meeting)

All potential scientific integrity violations (questionable pairs of abstracts) identified in this work were required to meet dual criteria: (a) their pairwise similarity scores exceed .57; and (b) verification by a domain expert. A domain expert subsequently classified violations by type defined above (see Fig. 1, middle right and Fig. 3, middle right).

Classifications of potential scientific integrity violations – into the aforementioned classes – were made by a domain expert (ethicist). Not every abstract was classified; indeed, classifying all 144,149 abstracts (1,235,085 pairwise similarity scores) was unfeasible. Instead, classifications were only considered for random samples of abstract pairs with similarity scores above .57. We arrived at this cutoff by considering the average similarity score (.27) and standard deviation (.12) across all 1,235,085 pairwise comparisons. Scores above .57 are approximately 2.5 standard deviations above the mean; consistent with most definitions of statistical outliers. Expert classifications were subsequently made for random samples of these abstracts with results being representative of the broader dataset (see Fig. 1, middle right).

To determine the appropriate sample size, we used the standard formula for one sample, dichotomous outcome:

$$ n=p\left(1-p\right){\left(\frac{Z}{E}\right)}^2 $$

where Z is the value from the standard normal distribution reflecting the confidence level that will be used (Z = 1.96) and E is the desired margin of error (E = .1). The value of p that maximizes p (1 - p) is .5 (*p* = 0.5). We find:

$$ 96.04=.5\left(1-.5\right){\left(\frac{1.96}{.1}\right)}^2 $$

Thus, when randomly sampling highly similar abstract pairs for domain expert classification we used a sample size of 100 (rounded up from 96).

Since we measured a dichotomous outcome with sufficiently large sample size, we approximated the binomial confidence interval using a normal distribution. All confidence intervals were calculated using the following standard formula:

$$ \mathrm{CI}=\pm \mathrm{Z}\sqrt{\frac{\hat{\mathrm{p}}\left(1-\hat{\mathrm{p}}\right)}{\mathrm{n}}} $$

### Two step calculation for estimating the rate of text similarity

We estimated the degree of similarity using a two-step calculation as follows: (a) calculate the fraction of all abstract pairs that are highly similar (*U*); and (b), calculate the fraction of highly similar abstracts that contain overlap (*V*). The frequency of abstracts with overlap was then estimated as the product: U*V.

## Results

### eTBLAST identifies highly similar abstract pairs in conference proceedings

**Fig. 4:**
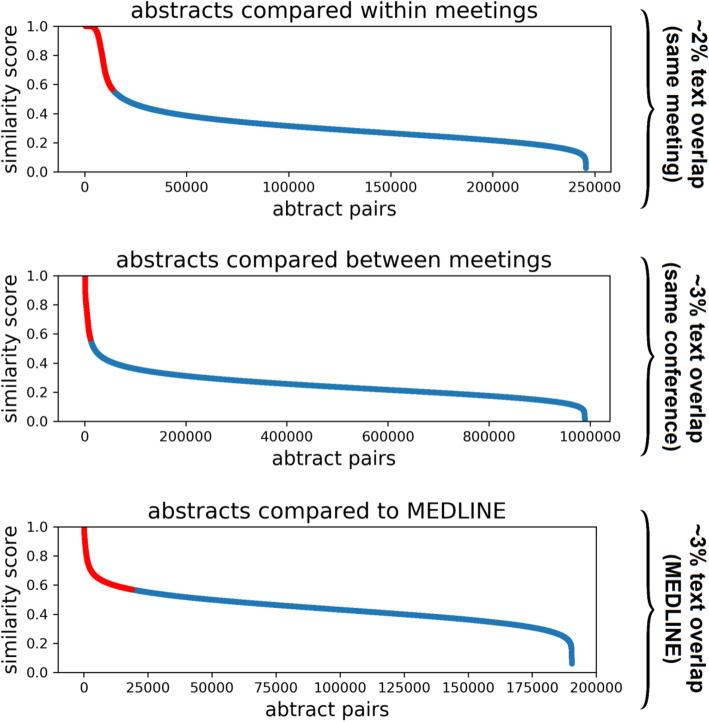
The distribution of similarity scores assigned by eTBLAST across all abstract pairs. Text similarity (eTBlast) identifies 20,857 abstracts similar to at least one other conference or published abstract. We used random samples of all such comparisons to estimate rates of text overlap – same meeting (2%); text overlap – same conference (3%); and text overlap - MEDLINE

Summary of our results are shown in Fig. 4 and Table 1. We used eTBLAST to assign similarity scores to 144,149 abstracts. Mean similarity score over all comparisons (1,235,085 abstract pairs) was 27 with a standard deviation of .12. Approximately 2% (24,365 total) of pairwise abstract similarity scores exceed .57 (2.5 standard deviations above the mean) which we used as a cut-off point to define text overlap. Note that the number of abstracts within highly similar pairs is not at all trivial. In fact, the number of abstracts within 24,365 pairs can range from 222 to 48,730. The lower bound is simply the result of 222 choose 2: $\frac{222!}{2!\left(222-2\right)!}>\mathrm{24,365}$. See methods for additional explanation. We found the abstracts within highly similar pairs number 20,857; approximately 14% of the 144,149 (Fig. 4, red lines). These 14% of overlaps are not necessarily instances of scientific misconduct; indeed, it is often acceptable to present similar abstracts at different meetings of the same conference or to present recently published work.

**Table 1:** Summary of main results

Highly similar abstracts identified by eTBLAST	5 samples of 100 abstract pairs for manual review	Abstracts verified by expert classification	Confidence interval (95%)	Example
**20,857 (out of 144,149)**	**Text overlap (same meeting)**	126 (12.6%)	10.6 to 14.8%	Figure S1
	**Text overlap (different meeting)**	223 (22.3%)	19.8 to 25.0%	Figure S2
	**Text overlap (Medline)**	238 (23.8%)	21.2 to 26.6%	Figure S3
	**Plagiarized**	9 (2.3%)	1.2 to 4.8%	Figure S5

### Extent and type of text overlap in conference abstracts

A domain expert then classified 1500 randomly sampled abstract pairs (approximately 3000 abstracts for text overlap): (a) within the same meeting, (b) between different meetings, and (c) between conference abstracts and MEDLINE abstracts. Briefly, we examined five random samples each of 100 highly similar (similarity score above .57 which is 2.5 standard deviations above the mean) abstract pairs and used expert classification to identify bona fide instances of potential misconduct (Fig. 1, middle right and Fig. 3, middle right). Sampling and evaluation was repeated for 3 types of misconduct (Fig. 1): Text overlap of abstracts 1) within the same meeting, 2) between different meetings and 3) between conference abstracts and MEDLINE abstracts. Rate of abstract plagiarism is estimated in the next section. See Table 1 for a summary of our main results.

Textual overlap between abstracts in the same meeting were quantified by sampling and evaluating abstract pairs presented concurrently at the same meeting. In 5 samples of 100 abstract pairs we verified 126 abstracts with textual overlap (out of 1000 abstracts in the randomly sampled pairs) or 13%.

Textual overlap between abstracts in different meetings of the same conference were quantified by sampling and evaluating abstracts pairs presented concurrently in different years (iterations) of the same conference. In 5 samples of 100 abstract pairs we verified 223 abstracts with textual overlap (out of 1000 abstracts in the randomly sampled pairs) or 22%.

Comparisons of conference abstracts with abstracts of journal articles published in MEDLINE were carried out. In 5 samples of 100 randomly selected pairs we verified 238 textually similar abstracts (out of 1000) or 24%. We emphasize that in some cases it may be acceptable to present abstracts from papers that are already published.

We used these results to estimate the degree of similarity using a two-step calculation (see methods for details). First, the fraction of all abstract pairs that are highly similar (*U*) was determined automatically by eTBLAST. Abstracts within highly similar pairs number 20,857 of the 144,149 (U = .14). We randomly sampled highly similar abstract pairs and manually identified 126 out of 1000 (V = .13). Thus, the estimated rate of text overlap - same meeting is:

$$ .14\ast .13\sim .02 $$

Rates of text overlap were quantified by sampling and manually evaluating abstracts pairs presented at different meetings of the same conference (i.e. similar abstracts presented in different years). Text overlap in all pairs totaled 223 out of 1000 (V = .22). Thus, the estimated rate of text overlap – same conference is:

$$ .14\ast .22\sim .03 $$

The interpretation of these results is not trivial. First, we reiterate that random samples were drawn only from the subset of highly similar abstract pairs; abstracts within those pairs represent 14% of the total (see above). Second, we reiterate that only a fraction of the randomly sampled abstracts were classified as having substantial text overlap accounting for approximately 2 and 3% of the total abstracts presented at scientific meetings (see Fig. 3 and Fig. 4).

Most of the randomly sampled abstracts were not classified as problematic; i.e. they were false positives. Fasle positives occurred for three reasons. First, HTML-scraped and PDF-scraped abstracts frequently contained aberrant HTML tags and document tags. These were not considered in subsequent analysis. Second, poster abstracts are frequently – and intentionally – paired with highly similar oral presentations; that is, some duplicate abstracts were from posters and oral presentations given by the same author/s. These types of duplications were classified as false positives. Third, most abstracts harboring potential textual overlap were fringe cases that were not deemed as being problematic.

### Rate of plagiarism

We checked for plagiarism by re-examining all 349 abstract pairs deemed to have significant textual overlap (Fig. 1, far right). In most cases (340 total), there was at least one author that was listed in both members of the pair. But, there were no overlapping authors in 9 of the cases thereby indicating ostensible plagiarism. However, these instances of plagiarism were debatable; for example, in several cases, identical passages were relegated to methods. We concluded that plagiarism between conference abstracts is rare: 0 to 3% (9 out of 349) of the sampled abstracts which in turn are 14% of the total. Thus, plagiarized conference abstracts account for 0% to .5% of all conference abstracts. Interestingly, investigations of published papers in Medline report similar findings. In 2008, there were 4.1 highly similar pairs of manuscripts per 1000 published papers in Medline and deposited in the Déjà vu database [50].

### Text overlap between conference abstracts and abstracts of subsequently published papers

**Fig. 5:**
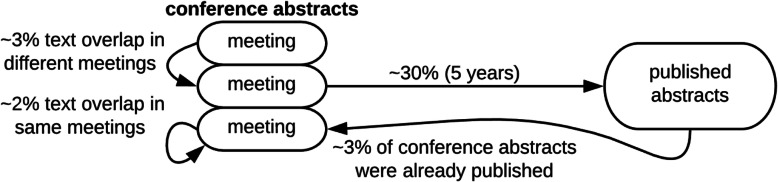
Estimated rates of conference abstract publication and duplication. Random samples of conference abstracts reveal approximately 3% were previously published and 3% were already presented. Comparatively more (30%) are published within 5 years

Our analysis suggests that approximately 3% of conference abstracts are recycled (near duplicates): i.e. recycled versions of previous conference abstracts and abstracts of published papers (Fig. 5). How does this compare to the abstracts from papers that have already been published? To shed light on this question we download 327,287 published abstracts from MEDLINE and used eTBLAST (see methods) to compare the 144,149 conference abstracts to the 327,287 published abstracts. We found 42,797 (out of 144,149) conference abstracts were highly similar (similarity score above .57) to at least one subsequently published abstract, i.e. the abstract is published after it appears in the proceedings of a scientific conference. We took into consideration the age of conference abstracts (most were presented in the last 5 years) and arrived at a simple interpretation: approximately 30% (42,797/144,149) of conference abstracts reach publication in 5 years.

### Case study of the American Association for Cancer Research annual meeting

To gain a better understanding of text overlap in conference abstracts, we examined those abstracts presented at meetings of the American Association for Cancer Research (AACR). Pairwise abstract similarity scores were computed within and between each collection of meeting abstracts: 5483 abstracts for 2015 and 5759 abstracts for 2017. The average similarity score for abstracts within each meeting is .31 with standard deviation .09. Between meeting similarity scores averaged .29 with standard deviation .09.

**Fig. 6:**
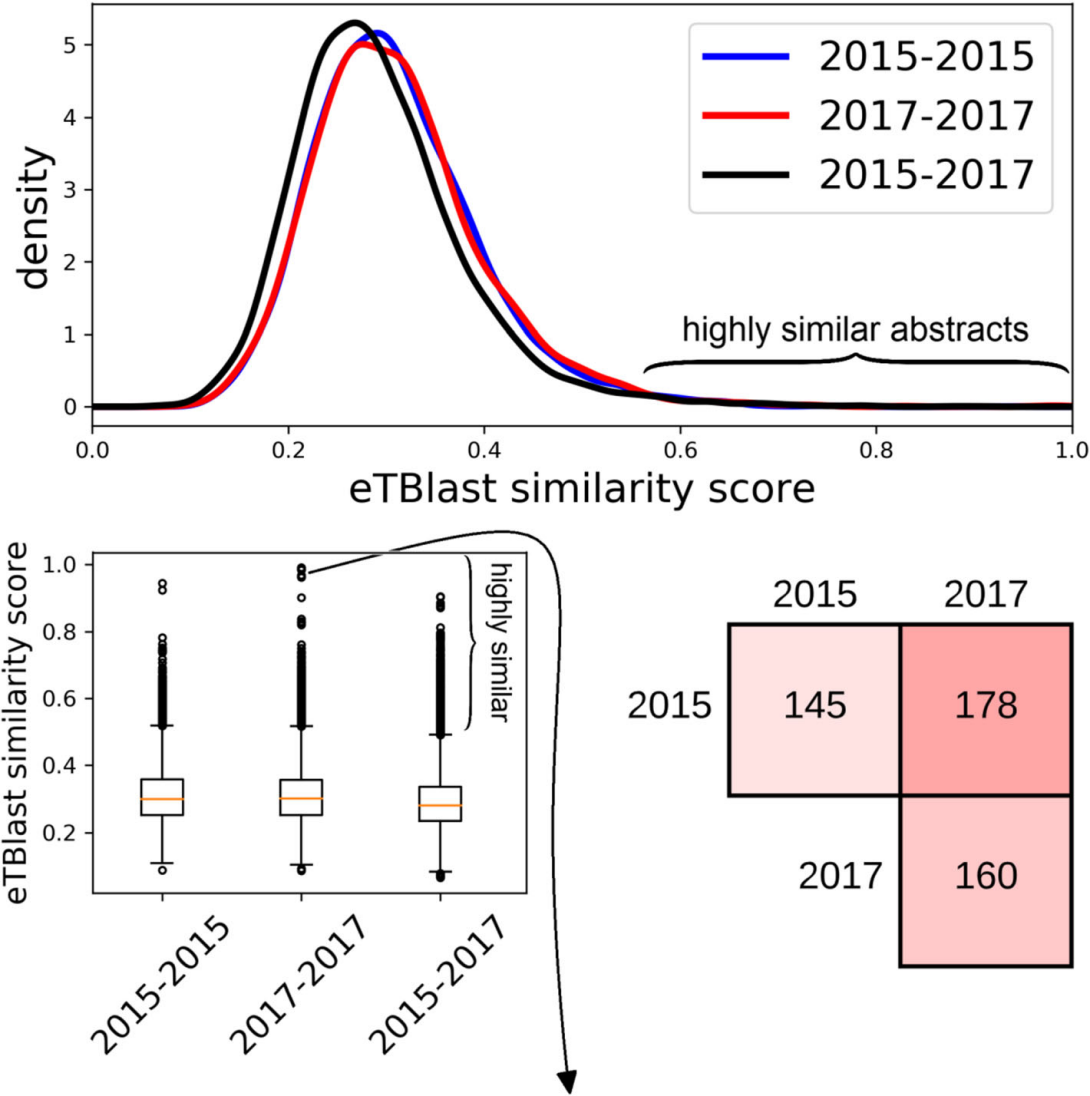
Summary of abstract similarity within and between meetings of the American Association for Cancer Research (AACR). top and middle left, distribution of pairwise similarity scores. Middle right, number of highly similar abstracts within and between the years 2015 and 2017. Bottom, abstracts #755 and #3139 in 2017 were identical but submitted under different titles

We used eTBLAST to identify highly similar (similarity score above .57) abstract pairs within and between meetings (Fig. 6). Within meeting pairs for 2015 and 2017 harbor 145 (for 2015) and 160 (for 2017) similar abstracts, respectively (Table 2). Pairs between those years (an abstract from 2015 paired with one from 2017) harbor 178 abstracts. Pairs of highly similar abstracts were reviewed by an ethicist who identified 36 and 53 instances of text overlap (ostensibly salami sliced) abstracts in 2015 and 2017, respectively (Table 2). While some of these instances are debatable, we identified several cases – 3 cases totaling 6 abstracts in 2017 – of identical abstracts submitted with different titles or different order of authors (Fig. 6). Manual review verified 90 of the potential 178 instances of abstract recycling (similar abstracts presented in 2015 and 2017). To the extent that AACR is representative of other large international conferences, the rate of text overlap between abstracts within meetings is approximately 1% (36 + 53 out of 5483 + 5759). This is slightly lower than the 2% estimate for all conferences (see above). The rate of text overlap from MEDLINE abstracts of previously published papers was approximately 1.5% (90 out of 5759).

**Table 2:** Summary results for case study of the American association for cancer research annual meeting

AACR meeting	Similar abstract pairs identified by eTBLAST	abstracts verified by expert classification	confidence interval (95%)
**2015**	145	36 (24.8%)	18.0 to 32.7%
**2017**	160	53 (33.1%)	25.9 to 41.0%
**2015/2017**	178	90 (50.6%)	43.0 to 58.1%

## Discussion

In our study, using eTBLAST and evaluation of one expert, we found 2% of textual overlap within the same meeting of biomedical conferences, 3% between meetings of the same conference, and 0.5% of plagiarism. Plagiarism of others’ work represents a serious ethical violation of long-established standards of scholarship, especially if the extent of copying rises to the level of research misconduct. Thus, given the results presented above, we believe that conference organizers need to exercise a greater degree of oversight of paper submissions received for consideration by, for example, using some of the popular fee-based text similarity platforms already available. Alternatively, free platforms, such as HelioBlast (previously known as eTBLAST) (http://etblast.org/ A free service maintained by Heliotext LLC) [50] with results compiled in the more recently developed EthicsDB (http://ethicsdb.org), described above, both of which are readily accessible, can be easily used to screen out potentially problematic submissions. It is our firm belief that meeting organizers have an ethical obligation to develop proper guidance in their call for papers and/or submission guidelines about what ethical parameters are expected and what steps will be followed should conference guidelines be grossly violated.

Obviously, there are some differences between the publishing and the conference platforms that may render some forms of copying more acceptable in conference presentations than they would in journal articles. For some pertinent discussion on these matters, we draw attention to a series of papers on the topic of ‘conference double-dipping’ [51–57], which can offer some guidance on the pros and cons of some of these ethically questionable practices. Many of the points that follow are derived from that set of papers.

Traditionally, the main purpose of scientific meetings is to provide an opportunity for the presentation of new findings and discussion of on-going investigations. Doing so allows researchers to quickly disseminate their results to interested audiences and receive the type of constructive criticism that can lead to meaningful improvements in methodology, analysis, and interpretation of results. Thus, double-dipping in conferences may be justifiable in some circumstances. For example, and particularly for early-career authors, feedback from one conference presentation can result in a revised product with clearer stated hypotheses, methods, and improved analyses, all of which can yield better-quality data and possibly even newer findings and/or interpretations the next time the work is presented to a different audience [51–53]. On the other hand, many conference meetings have a limited number of submission slots. Thus, acceptance of a duplicate submission, even a substantially revised one, means that another colleague has been denied the opportunity to present his/her novel work [52].

One situation that may justify the types of substantial amounts of textual overlap that we observed in our data occurs with ‘salami presentation’, the segmentation of a large data set into smaller units. Most often individual slots for paper sessions are between 15 and 20 min per paper with barely 5 min for questions or comments. When presenting the results of a large, complex study, such short intervals are typically less than adequate to describe the most important findings, let alone their totality. A similar situation occurs with posters: There is only so much material that one can fit into a poster. However, literature reviews, methods, and perhaps some discussion material will be similar across salami-sliced posters. Consequently, in some cases and the absence of relevant guidance, salami-sliced presentations are not only unavoidable, they may even be desirable. And, in fact, it is not uncommon in poster sessions to see a series of papers coming from the same lab and often sharing some of the same literature review, methods, and some or all authors, describing various facets of a complex set of experiments. Ideally, all of the individual components of these complex projects would have been presented together in a single presentation, but typical space and/or time limitations cannot accommodate these large projects. Surely, such situations should not discourage researchers from submitting serial papers, as long as these comply with conference submission guidelines and there is full transparency in all presentations about how these separate papers are inter-related. Such an approach is especially important in situations in which the exclusion of some of the ‘salami papers’ might give the audience an incomplete or misleading appreciation of the research effort [55].

All of these considerations lead us to strongly urge conference organizers to develop comprehensive guidance for conference submissions that address the various types of potentially problematic submission patterns containing substantial textual overlap: ‘present-little or no revision-present again’ vs. some version of salami presentation, such as ‘present-refine/add data-present again’ [52]. We recognize that each conference may have different sets of goals, different types of audiences, and that their meetings can vary in competitiveness in ways similar to high vs. low impact journals. However, perhaps such forms of double-dipping, particularly the ethically questionable ‘present-no revision-present again’ do not seem to make much sense at a time when governmental funding for science, as well as most institutional conference travel budgets, have become so competitive. Thus, in the same way that duplicate grant proposals that have received funding result in the denial of funding for other worthy, original research [58], travel funding for double-dipped presentations means that other potentially valuable research might not be disseminated.

The analysis reported herein confirms that the level of plagiarism at scientific meetings is very similar to what we reported for peer-reviewed publications before the wide-spread use of tools by journals to identify suspect manuscript submissions. We found more textual overlap suggesting plagiarism than self-plagiarism, which was somewhat surprising in light of earlier studies of conference submissions [40–43]. It is possible that text recycling in conference papers is simply just a more acceptable, perhaps even expected, practice in certain disciplines within the social sciences [54, 57] relative to those in the biomedical sciences. One possible explanation for the finding regarding plagiarism may be that those tempted to cross an ethical line refrain from doing so at meetings where there is face-to-face direct contact with those you may have “borrowed” from. Our data support that conjecture in two ways. At smaller meetings among smaller research communities there is less similarity among abstracts; and, across all meetings, there was no evidence of any plagiarism where the material was taken from meeting abstracts. We suggest that a more permanent, homogeneous archive of conference proceedings is needed to facilitate research on the etiology of scientific misconduct, novelty, and breakthroughs. To the end, our database of results (ethicsdb.org) should not be overlooked.

One obvious limitation with the present study is that textual overlap detected by our methods is confined to the conference abstracts and not the actual presentations themselves. Thus, it is conceivable that in some cases (many?) greater amounts of overlap are found in the actual presentations themselves. In a similar vein, and as noted by a referee, although our comparisons spanned 3 meeting years, it is possible, even likely, that there were instances of text overlap that occurred across longer time spans between conference abstracts. In sum, and given that previous work using a similar methodology with Medline abstracts resulted in 56 retractions of the scientific literature [35] (plus dozens more not recorded in Medline [59]) within months following the initial publication [34], we are confident that our data represents a good indication, albeit it a conservative one, of significant overlap in actual presentations.

## Conclusions

Scientific progress depends on accurate and reliable information exchange. Dissemination of research findings represents the seed for future scientific discoveries and validation for existing observations and theories. There has been substantial progress injecting research integrity principles into the peer-reviewed literature, however, it is also important for these principles to be similarly applied to the other major exchange modality, the scientific meeting. This analysis indicates that textual overlap in abstracts of papers presented at scientific meetings is one-tenth that of peer-reviewed publications, yet the plagiarism rate is approximately the same as previously measured in peer-reviewed publications. This latter finding underscores a need for monitoring scientific meeting submissions – as is now done when submitting manuscripts to peer-reviewed journals – to improve the integrity of scientific communications.

## Supplementary Information

**Additional file 1: Figure S1.** An example of text overlap – same meeting. Both abstracts were presented at the same meeting: 2014 European Association for the Study of Obesity. The abstracts have overlapping authors, and both were presented as posters. Only minor, insignificant changes have been made to the methods, results, and conclusions. Measurements from the results sections in each abstract are identical. Both were presented as posters. **Figure S2.** An example of text overlap – same conference. The left abstract was presented at the American Society of Tropical Medicine and Hygiene, 2013. The right abstract was presented at the same conference in 2016. Title and results are identical with minor changes to the abstractive narrative. Abstracts share at least one overlapping author. **Figure S3.** An example of text overlap - MEDLINE. The left abstract was published in the June 2008 issue of *Parasitology International*. Four year later (in 2012) the same abstract was presented as a poster at the American Society of Tropical Medicine and Hygiene. **Figure S4.** An example of false-positives. Both abstracts were presented at the same meeting: the 2018 International Association for the Study of Lung Cancer. According to eTBLAST, these abstracts have a similarity score of .998. A domain expert classifies these as false-positives because one was presented as a poster (right), the other was presented as a talk (left). Only posters were considered in our analysis. We did not consider workshops, plenary talks, or keynotes. **Figure S5.** An example of a putative plagiarism. The right abstract was published in 2013 (Kang JW, Song HG, Yang DH, Baek S, Kim DH, Song JM, Kang DH, Lim TH, Song JK. Association between bicuspid aortic valve phenotype and patterns of valvular dysfunction and bicuspid aortopathy: comprehensive evaluation using MDCT and echocardiography. JACC Cardiovasc Imaging. 2013 Feb;6(2):150–61. doi: https://doi.org/10.1016/j.jcmg.2012.11.007. PMID: 23489528.) The left abstract was presented in the 2018 Society of Thoracic Surgeons, abstract 14,001. According to eTBLAST, these abstracts have a similarity score of .75. Sentences with particularly significant similarity is underlined.

## Data Availability

All data is available in the website/database we created for this project, www.ethicsdb.org
