# Supplementary material for: Estimating the prevalence of text overlap in biomedical conference abstracts
Source: Res Integr Peer Rev. 2021 Feb 1;6:2. doi: 10.1186/s41073-020-00106-y (PMC7849107; doi:10.1186/s41073-020-00106-y)
Supplement: Supplementary file 1 — Additional file 1: Figure S1. An example of text overlap – same meeting. Both abstracts were presented at the same meeting: 2014 European Association for the Study of Obesity. The abstracts have overlapping authors, and both were presented as posters. Only minor, insignificant changes have been made to the methods, results, and conclusions. Measurements from the results sections in each abstract are identical. Both were presented as posters. Figure S2. An example of text overlap – same conference. The left abstract was presented at the American Society of Tropical Medicine and Hygiene, 2013. The right abstract was presented at the same conference in 2016. Title and results are identical with minor changes to the abstractive narrative. Abstracts share at least one overlapping author. Figure S3. An example of text overlap - MEDLINE. The left abstract was published in the June 2008 issue of Parasitology International. Four year later (in 2012) the same abstract was presented as a poster at the American Society of Tropical Medicine and Hygiene. Figure S4. An example of false-positives. Both abstracts were presented at the same meeting: the 2018 International Association for the Study of Lung Cancer. According to eTBLAST, these abstracts have a similarity score of .998. A domain expert classifies these as false-positives because one was presented as a poster (right), the other was presented as a talk (left). Only posters were considered in our analysis. We did not consider workshops, plenary talks, or keynotes. Figure S5. An example of a putative plagiarism. The right abstract was published in 2013 (Kang JW, Song HG, Yang DH, Baek S, Kim DH, Song JM, Kang DH, Lim TH, Song JK. Association between bicuspid aortic valve phenotype and patterns of valvular dysfunction and bicuspid aortopathy: comprehensive evaluation using MDCT and echocardiography. JACC Cardiovasc Imaging. 2013 Feb;6(2):150–61. doi: https://doi.org/10.1016/j.jcmg.2012.11.007. PMID: 23489528.) The left abstra [file 41073_2020_106_MOESM1_ESM.docx]

**Supplementary material**


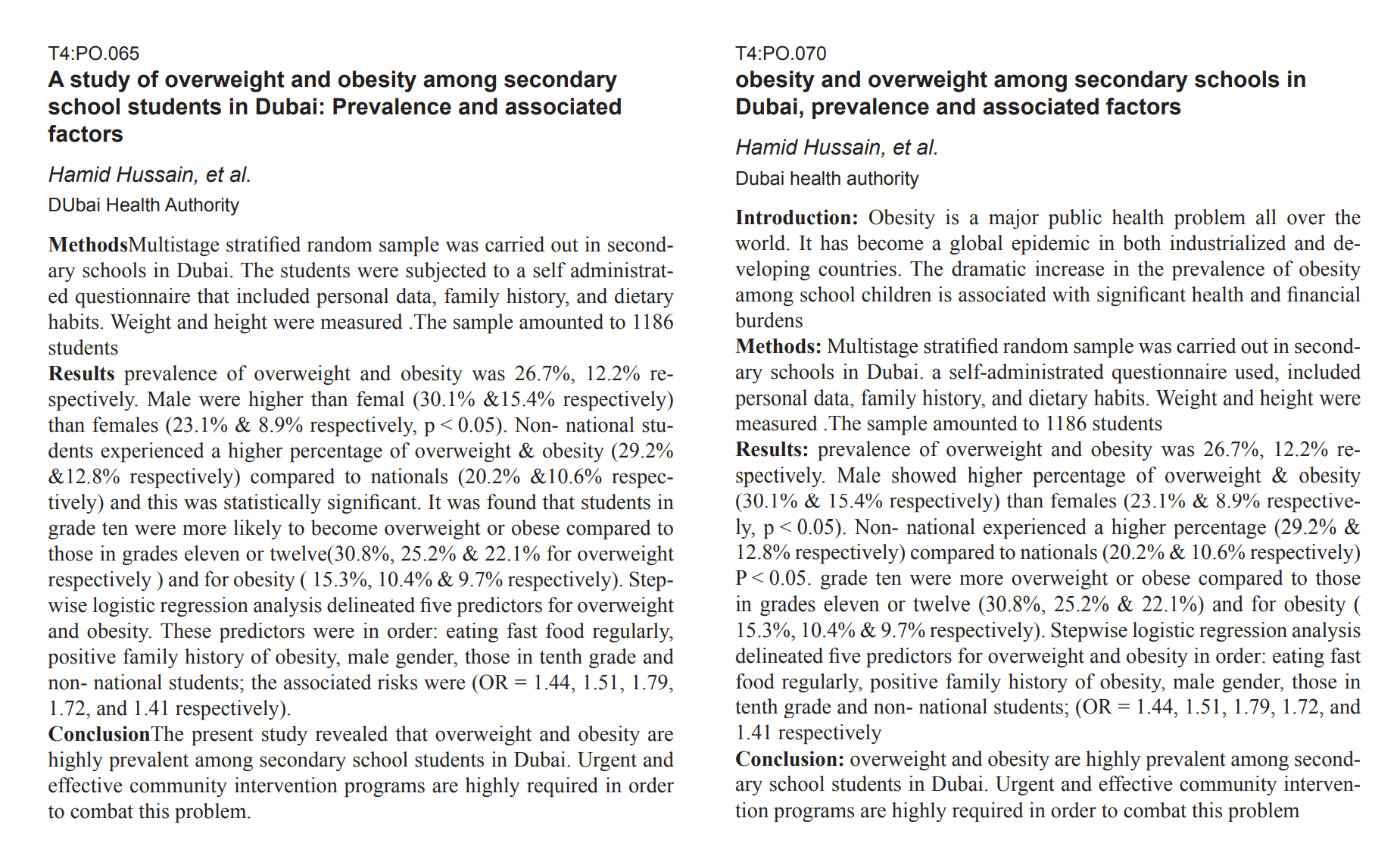


**Figure S1 – An example of text overlap – same meeting.** Both abstracts were presented at the same meeting: 2014 European Association for the Study of Obesity. The abstracts have overlapping authors, and both were presented as posters. Only minor, insignificant changes have been made to the methods, results, and conclusions. Measurements from the results sections in each abstract are identical. Both were presented as posters.


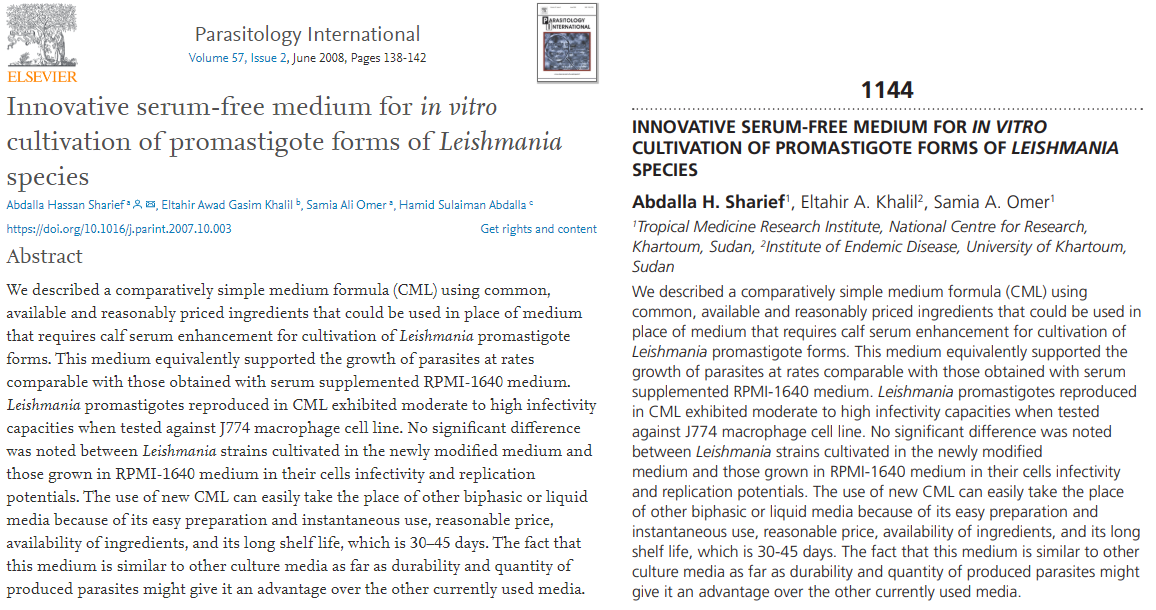


**Figure S3 – An example of text overlap - MEDLINE.** The left abstract was published in the June 2008 issue of *Parasitology International*. Four year later (in 2012) the same abstract was presented as a poster at the American Society of Tropical Medicine and Hygiene.


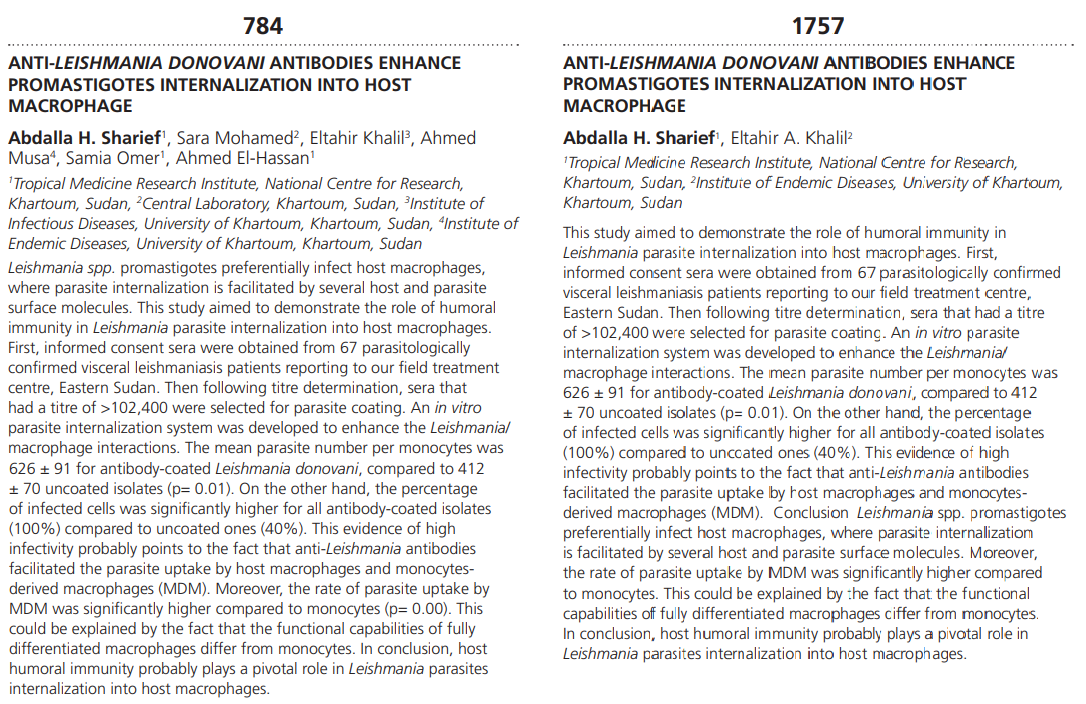


**Figure S2 – An example of text overlap – same conference.** The left abstract was presented at the American Society of Tropical Medicine and Hygiene, 2013. The right abstract was presented at the same conference in 2016. Title and results are identical with minor changes to the abstractive narrative. Abstracts share at least one overlapping author.


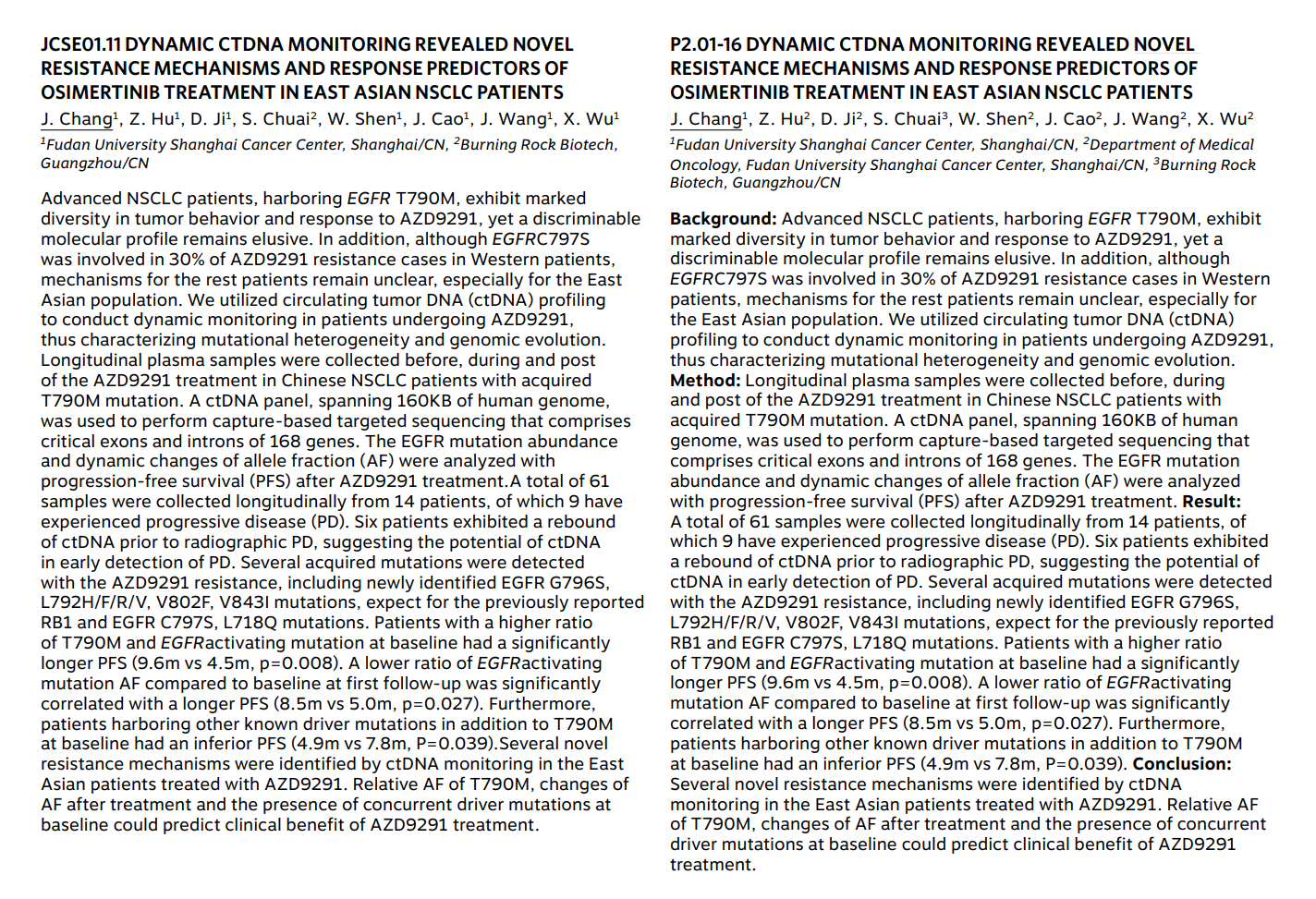


**Figure S4 – An example of false-positives.** Both abstracts were presented at the same meeting: the 2018 International Association for the Study of Lung Cancer. According to eTBLAST, these abstracts have a similarity score of .998. A domain expert classifies these as false-positives because one was presented as a poster (right), the other was presented as a talk (left). Only posters were considered in our analysis. We did not consider workshops, plenary talks, or keynotes.


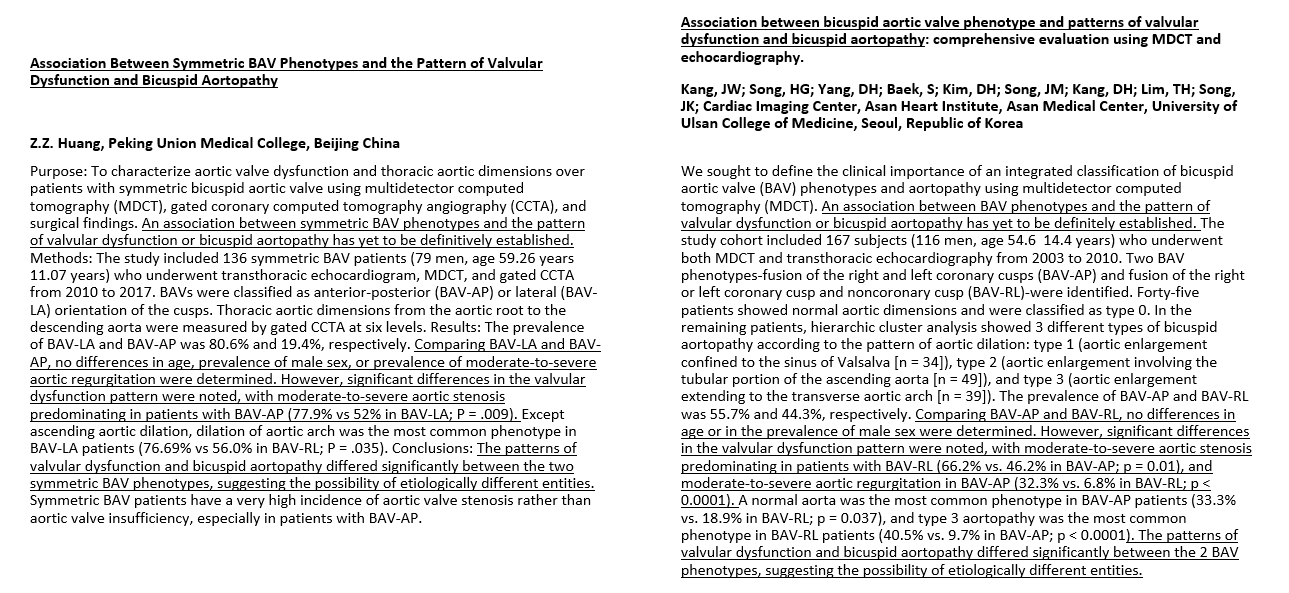


**Figure S5 – An example of a putative plagiarism.** The right abstract was published in 2013 (Kang JW, Song HG, Yang DH, Baek S, Kim DH, Song JM, Kang DH, Lim TH, Song JK. Association between bicuspid aortic valve phenotype and patterns of valvular dysfunction and bicuspid aortopathy: comprehensive evaluation using MDCT and echocardiography. JACC Cardiovasc Imaging. 2013 Feb;6(2):150-61. doi: 10.1016/j.jcmg.2012.11.007. PMID: 23489528.) The left abstract was presented in the 2018 Society of Thoracic Surgeons, abstract 14001. According to eTBLAST, these abstracts have a similarity score of .75. Sentences with particularly significant similarity is underlined.
